# Malnutrition as a Risk Factor for Falls and Respiratory Infections in Nursing Home Residents

**DOI:** 10.7759/cureus.95929

**Published:** 2025-11-02

**Authors:** Julijana Pelivan, Ivana Sovic, Bojan Miletic, Lejla Jelovica, Gordana Starcevic-Klasan, Davor Horvat

**Affiliations:** 1 Department of Clinical Medical Sciences, Faculty of Health Studies, University of Rijeka, Rijeka, HRV; 2 Department of Basic Medical Sciences, Faculty of Health Studies, University of Rijeka, Rijeka, HRV; 3 Department of Internal Medicine, General Hospital Ogulin, Ogulin, HRV; 4 Department of Internal Medicine, General Hospital Karlovac, Karlovac, HRV

**Keywords:** elderly falls, malnutrition, public health care, respiratory infections, elderly

## Abstract

Background

The aim of this study was to examine the prevalence of malnutrition and its association with the frequency of falls and respiratory infections among older adults residing in Croatian nursing homes.

Materials and methods

The study included 148 participants, 112 (75.7%) women and 36 (24.3%) men, aged 65 years and older, living in the nursing homes in Rijeka and Opatija. The Mini Nutritional Assessment-Short Form (MNA-SF) and data from the medical records were used for data collection.

Results

The study showed that seven (4.7%) of the older adults living in nursing homes were malnourished, while 53 (35.8%) were at risk of malnutrition. It was found that participants with malnutrition and nutritional risk were more likely to develop respiratory infections (r=-0.37). No correlation was found between malnutrition and the frequency of falls in older adults (r=0.01).

Conclusion

Malnutrition and the risk of malnutrition are common problems in older adults in nursing homes, requiring regular monitoring and timely intervention. The results confirmed the association between malnutrition and respiratory tract infections, while also highlighting the possibility of co-occurrence of obesity and malnutrition, which is often overlooked.

## Introduction

Nutritional status is a crucial component of a healthy life, as it depends on individual nutrient requirements and their intake [1]. An unbalanced intake of nutrients leads to a state of malnutrition. Due to the global increase in the proportion of the elderly population, the importance of malnutrition in older adults is becoming increasingly significant. The physiology of malnutrition in older adults begins with the process of biological aging, which increases susceptibility to acute and chronic diseases and conditions that impair nutrient absorption, metabolism, and immune function [2,3]. Odor and taste disturbances, polypharmacy, and gastrointestinal tract abnormalities in older adults can lead to delayed gastric emptying and suppressed intestinal motility, resulting in early satiety and decreased appetite [2,3]. Reduced nutrient absorption leads to an energy deficit, which affects the various organ systems. Sarcopenia, osteoporosis, and muscle weakness are consequences of malnutrition that impair the functionality of the musculoskeletal system and further increase the risk of falls in older adults [3].

According to a study by Mziray et al., approximately 30% of the geriatric population experienced falls, with the highest risk observed among malnourished older adults living in nursing homes [4]. Malnutrition and a low body mass index (BMI) are associated with an increased risk of falls, with malnourished older adults having an eightfold higher risk of falling than those with no evidence of malnutrition [5]. In addition to affecting the mechanics themselves, malnutrition weakens immune function, resulting in greater exposure and susceptibility to infectious and other inflammatory diseases. In older age, malnutrition is associated with a higher incidence of urinary tract infections, pneumonia, and tuberculosis, making wound healing more difficult and consequently prolonging hospitalization. Malnutrition also reduces the body's response to vaccines and is associated with reduced production of lymphocytes and cytokines, which affects the immune response [6-8].

Social factors also have a significant impact on the consequences of malnutrition, which further emphasizes the problem in the context of institutional care. According to data from the study by Ulger et al., of 534 older adults living in nursing homes in Turkey, 85 (15.9%) were malnourished and 286 (53.6%) were at the risk of malnutrition [9]. In Croatia, about one-fifth (22.8%) of the total sample of 123 elderly nursing home residents were at a risk of malnutrition [10]. Older adults who are hospitalized or immobilized are particularly affected by malnutrition. In these individuals, the lack of physical activity often leads to reduced food intake, difficulty swallowing, impaired nutrient absorption, loss of muscle mass, and, consequently, malnutrition [11,12]. In addition, long-term immobility or limited mobility leads to progressive muscle weakness, increased frequency of falls, suppression of immune mechanisms, and increased susceptibility to infections [13,14]. This sets in motion a downward spiral. Despite all these facts and though the Croatian Guidelines for Nutrition of the Elderly have been published, the municipalities do not adhere to them [15].

A particular limitation in current research is the lack of studies focusing specifically on older adults living in care homes who are independently mobile and can move around without assistance, or who are partially mobile with the use of aids such as walkers or canes. To address these under-researched areas, this study aims to investigate the prevalence of malnutrition and nutritional risk, as well as their association with falls and respiratory infections, in older mobile people living in nursing homes in Rijeka and Opatija. These cities are located in Primorje-Gorski Kotar County, a coastal region in western Croatia with a growing elderly population and several long-term care facilities, making them representative of the study of institutionalized older people in this area.

## Materials and methods

Participants

This cross-sectional study was conducted between January 2024 and March 2025 in nursing homes located in Rijeka and Opatija, Primorje-Gorski Kotar County, Croatia. A total of 148 residents aged 65 years and older (112 women and 36 men) participated. Participants were consecutively recruited based on the following inclusion criteria: age ≥65 years, permanent residence in a nursing home, and ability to provide informed consent. Residents were excluded if they had an acute illness affecting nutritional status, were in a terminal stage, or were unable to give informed consent.

Participants were divided into four age groups (65-70, 71-80, 81-90, and over 90 years) to reflect common geriatric categories and differences in functional and nutritional status across the older adult lifespan.

The study locations (Rijeka and Opatija) were chosen to represent urban and suburban nursing home populations within the Primorje-Gorski Kotar County, providing a diverse sample of older adults living in institutional care. Data collection was performed through face-to-face interviews using the Mini Nutritional Assessment-Short Form (MNA-SF) [16], complemented by a review of medical records for falls and respiratory infections over the previous year.

The sample size of 148 participants was determined based on feasibility and available residents meeting the inclusion criteria, ensuring adequate representation across age groups and mobility levels. This study was designed to ensure replicability by clearly documenting all procedures, instruments, and data collection steps.

Testing procedures and data collection

The standardized MNA-SF questionnaire was used to assess nutritional status [16]. The development of the Mini Nutritional Assessment (MNA) questionnaire began in 1989, and it was validated and published in 1994 [17,18]. A shorter version, the MNA-SF questionnaire, was published in 2001, while the revised version was published in 2009 [18,19]. According to the Nestlé Nutrition Institute's terms of use, the MNA and MNA-SF questionnaires are available for download for non-commercial clinical use only [20]. In this study, the original and standard MNA-SF questionnaire, translated into Croatian, was used directly without any modifications to assess the nutritional status of older adults in residential care. The questionnaire was administered face-to-face by trained investigators, who guided participants through each item to ensure accurate responses. All procedures were strictly followed according to the original scoring and interpretation guidelines, in accordance with the license agreement from the Nestlé Nutrition Institute. The questionnaire was completed by the investigator together with the participants. Responses were scored on a scale from zero to three. The maximum total score after completing the questionnaire was 14 points. The results were categorized into point intervals. A score ≥12 represented a normal nutritional status. A score between eight and 11 represented people at risk of malnutrition. Participants who scored less than eight points were categorized as malnourished [16]. 

Anthropometric measurements were conducted to calculate BMI. Height was measured with participants standing upright, barefoot, and with heels together. Body weight was measured with participants wearing light clothing. Both measurements were taken using a medically-calibrated digital floor scale with a stadiometer (ADE M319660-002, ADE Germany GmbH, Hamburg, Germany). BMI was calculated as weight in kilograms divided by height in meters squared (kg/m²). During measurement, care was taken to ensure the participants were stable and able to stand safely, heavy clothing and shoes were removed, and all measurements were recorded twice to ensure accuracy.

Data on the incidence of falls and respiratory infections in the previous year were retrospectively collected from participants' medical records. This research was conducted as part of the project entitled “Socio-demographic and functional parameters as determinants of frailty syndrome in older people in nursing homes”. The principal investigator of the project is Bojan Miletic, one of the authors of this article, employed at the University of Rijeka, Croatia. Ethical approval for the main project and the present study, including access to participants’ medical records, was obtained from the Professional Councils of the Nursing Homes Kantrida (approval no. 01-1795/24) and Volosko (approval no. 2156-07003/U2-3-2024-122), which in Croatia serve as the ethical oversight bodies for research conducted in nursing homes. No additional ethical approval was required for the present study, as it used data collected under the original approved project. All procedures were conducted in accordance with Croatian laws on data protection and the principles of the Declaration of Helsinki.

Statistical analysis

The demographic data of the participants were described using descriptive statistics. The values from the MNA-SF questionnaire were recorded on a scale, and the number of participants in each category was presented as a percentage. BMI values, height, body weight, and MNA-SF values were presented as means and standard deviations. Differences in nutritional status between the four age groups of participants were tested using a non-parametric Kruskal-Wallis analysis of variance (ANOVA) test with post-hoc analysis. Pearson correlation was used to examine the relationship between the following variables: Nutritional status and incidence of respiratory infections, nutritional status and BMI values, and nutritional status and incidence of falls in older adults. The significance level of the differences was set at p>0.05 (95%). Data analysis was performed using IBM SPSS Statistics for Windows, Version 25 (Released 2017; IBM Corp., Armonk, New York, United States) and Jeffreys's Amazing Statistics Program (JASP), version 0.17.2.1 (Department of Psychological Methods, University of Amsterdam, Amsterdam, Netherlands).

## Results

A total of 148 people were surveyed as part of this study, whose basic socio-demographic data are listed in Table 1.

**Table 1:** Basic sociodemographic characteristics of participants N: number of participants; %: percentage; t: value for testing the significance of the Pearson correlation coefficient; p: probability value.

Characteristics	N	%	t	p
Gender				
Men	36	24.3		
Women	112	75.7		
Age (years)				
65-70	8	5.4	26.289	0.000
71-80	41	27.7		
81-90	73	49.3		
>90	26	17.6		

Of these, 36 (24.3%) were men and 112 (75.7%) were women. The average age of the respondents was 83.6 ± 7.5 years. The analysis revealed a significant difference between the age groups (t=26.289, p=0.000). Participants were divided into four age groups (65-70, 71-80, 81-90, and over 90 years) to allow a detailed analysis of trends in nutritional status, falls, and respiratory infections across the older adult's lifespan. Although the group sizes were unequal, non-parametric statistical tests (Kruskal-Wallis) were applied, which do not require equal group sizes, ensuring robust comparisons.

The average BMI among the participants was 24.6 ± 3.4 kg/m^2^, placing them within the normal weight range (Table 2).

**Table 2:** The average values for BMI, height, weight, and MNA score BMI: Body Mass Index; MNA: Mini Nutritional Assessment; SD: Standard Deviation.

	Mean	SD
BMI	24.6	3.4
Height (cm)	165.3	9.5
Weight (kg)	68.1	11.3
MNA score	11.75	4.1

Table 3 shows a statistically significant difference in two MNA score categories (MNA scores of eight to 11 and 12 to 14) by gender.

**Table 3:** Values of MNA scores by gender MNA scores: Mini Nutritional Assessment scores; p: probability value.

MNA scores	Men	Women	p
0-7	5	2	0.089
8-11	15	38	0.002
12-14	16	72	0.000

The majority of participants achieved a normal MNA score; seven (4.7%) participants were categorized as malnourished, while a further 53 (35.8%) participants were found to be at risk of malnutrition. Risk of malnutrition was significantly more common in women (p=0.002). In addition, the same analysis showed that malnutrition was significantly more common in men (p=0.000). The analysis showed a significant difference in MNA scores between age groups (p=0.011). The post-hoc analysis showed the greatest difference between participants aged 61-70 years and those aged 90+ years (p=0.028), indicating a trend in malnutrition rates among older adults.

Further analysis revealed significant differences in mobility levels based on the gender of the participants (p<0.001) (Table 4).

**Table 4:** Comparison of the mobility level of the participants by gender p: probability value.

Mobility level	Men	Women	p
Move independently with assistance	21	57	0.000
Move independently without assistance	15	55	0.000

In the entire sample, 21 (14.1%) men and 57 (38.5%) women reported needing assistance with moving, which together account for 78 (52.6%) participants of the total sample. The post-hoc analysis shows that the biggest difference is between the participants who move independently without assistance (p=0.018).

In the next step of the statistical analysis, we explored the relationship between MNA scores and BMI values. Our analysis yielded a Pearson correlation coefficient of (r=-0.39), indicating a significant negative relationship. This evidence suggests that individuals with lower MNA scores tend to have higher BMI values (Table 5).

**Table 5:** Average BMI values in different MNA score groups MNA: Mini Nutritional Assessment; BMI: Body Mass Index; r: Pearson correlation coefficient.

MNA scores	Average BMI values	r
0-7	26.6	- 0.39
8-11	24.2	
12-14	23.2	

Moreover, the statistical analysis showed no significant differences between the participants' age or gender and the occurrence of respiratory infections. However, it did reveal a noteworthy negative correlation (r=-0.37) between the number of respiratory infections and instances of malnutrition (Table 6).

**Table 6:** The average number of respiratory infections in different MNA score groups MNA scores: Mini Nutritional Assessment Scores; r: Pearson correlation coefficient.

MNA scores	The average number of respiratory infections	r
0-7	1.76	- 0.37
8-11	1.64	
12-14	1.56	

Research indicates a significant correlation between the frequency of falls and both age and mobility. A positive correlation (r=0.54) was established between age groups and the average frequency of falls. This indicates that older participants tend to fall more frequently, and vice versa (Table 7).

**Table 7:** The average number of falls relating to the age groups of participants r: Pearson correlation coefficient.

Age groups	Average number of falls	r
65-70	1.00	0.54
71-80	1.09	
81-90	1.23	
>90	1.81	

Furthermore, a statistically significant correlation (r=0.44) was established between mobility levels and the frequency of falls. Data obtained indicates that more mobile individuals tend to fall less frequently, while those with lower mobility fall more often (Table 8).

**Table 8:** The average number of falls relating to the participants' mobility levels r: Pearson correlation coefficient.

Mobility level	Average number of falls	r
Move independently with assistance	1.29	0.44
Move independently without assistance	1.00	

At the same time, the analysis did not show any relationship between the frequency of falls and malnutrition (r=0.01).

## Discussion

The results of the study showed that seven (4.7%) of the older adults living in nursing homes were malnourished. While these results may seem positive, when taking into account not only the participants categorized as malnourished but also those at risk of malnutrition, the overall percentage increases to 43.2% of the sample. Similar results were found in a study conducted in Turkey, where the prevalence of malnutrition in the sample of 773 residents was 8.4%, while 37% of older adults were at risk of malnutrition [21].

In our study, almost half of the participants faced either malnutrition or nutritional risk, highlighting a substantial proportion of nursing home residents who require targeted attention. This underscores the importance of early detection of malnutrition and continuous monitoring in older adults, as deterioration can occur rapidly. The "at risk of malnutrition" group is particularly dynamic and clinically significant, as individuals in this category may quickly progress to manifest malnutrition due to short-term triggers such as acute illness, depression, cognitive decline, or social isolation [22,23]. These individuals often show changes in body composition, such as loss of muscle mass and impaired micronutrient intake, which may not be immediately reflected in BMI measurements [24]. Timely nutritional interventions, dietary counseling, oral nutritional supplements, exercise programs, and strategies to address social and psychological barriers, can prevent progression from nutritional risk to malnutrition and reduce associated complications such as falls, infections, and functional decline [22,25].

The European Society for Clinical Nutrition and Metabolism (ESPEN) guidelines strongly recommend proactive management of older adults at risk, emphasizing regular screening with validated tools such as the MNA-SF, integration of multidisciplinary care teams, and monitoring of both macro- and micronutrient intake [22,26]. Given the high prevalence of individuals at nutritional risk, nursing homes need to implement structured nutrition care pathways, ensuring early identification and individualized interventions to maintain health, functional independence, and quality of life in older residents [22]. In Norway, malnutrition was associated with lower BMI values [27]. In contrast, the results of our study showed a negative correlation between malnutrition and BMI. Older adults with lower MNA-SF values had higher BMI values. This result may seem illogical, as malnutrition is usually associated with a lower BMI. However, it could indicate a poorer quality diet with a lack of micronutrients and proteins. It could also indicate a loss of muscle mass despite excess body fat, a condition known as sarcopenic obesity. Finally, it should be noted that the MNA-SF takes into account changes that have occurred within the last three months. Recent admission to a nursing home, emotional stress, or acute illness before admission could influence the occurrence of a high BMI combined with a low MNA score as measured by this questionnaire. The MNA-SF assesses recent changes and nutritional risk, while the BMI only reflects body mass index based on total body weight, meaning that a person may appear "well nourished" based on their weight while also being at high nutritional risk. This means that the MNA-SF score reflects only the current status, which can change rapidly and must be monitored to prevent complications.

This is particularly important when considering the age of the participants. The results of our study show that the prevalence of malnutrition increases with age and is highest in participants aged 90 years and older compared to those aged 65 to 70 years. This age-related increase in malnutrition may be explained by physiological changes such as decreased appetite, reduced muscle mass, impaired nutrient absorption, and higher prevalence of comorbidities in the oldest-old population. In addition to age, gender differences were also observed. While studies from Norway and Turkey have reported higher malnutrition rates among older women [21,27], our study found a greater number of malnourished men, particularly in the oldest age groups, which also suggests a possible role of regional and sociodemographic characteristics.

One of the key elements to reduce complications in older adults is to maintain mobility. In our study, there was a significant negative correlation (r=-0.44) between the degree of mobility and the incidence of falls, suggesting that more mobile individuals tend to fall less frequently, while those with less mobility fall more frequently. The association between immobility or reduced mobility and a higher incidence of falls in hospital has been well documented in previous studies, such as the study by Kissane et al. [28]. This is particularly relevant for older adults as they age. For this reason, our study focused only on mobile participants, an area that remains under-researched. The average number of falls for mobile participants was in our study 1.00, compared to 1.29 for participants with limited mobility. The highest average number of falls was observed in participants over 90 years of age, with an average of 1.81 falls, which represents a significantly higher frequency of falls compared to younger age groups.

However, the analysis conducted in our study showed no significant correlation between malnutrition and the frequency of falls (r=0.01). This finding contrasts with several studies, including research from the Netherlands, which reported that malnourished older adults were more likely to experience falls, confirming a link between poor nutritional status and fall risk [29]. Several factors may explain this discrepancy. First, all participants in our study were mobile to varying degrees, including those who were independently mobile or required minimal assistance. Mobility is a strong determinant of fall risk, and it is possible that adequate physical function mitigated the impact of malnutrition on falls in our sample. In addition, the relationship between malnutrition and falls may be influenced by factors such as sarcopenia, polypharmacy, or cognitive decline, which were not directly assessed in this study. Our measure of malnutrition primarily reflects recent nutritional risk, whereas the risk of falls may be affected by long-term nutritional status and cumulative deficits. The co-occurrence of obesity and malnutrition can obscure the relationship between malnutrition and fall risk. Individuals may have a normal or high BMI while experiencing muscle weakness and functional decline, which could contribute to falls independently of MNA-SF classification [30]. These findings highlight the complex nature of fall risk in older adults.

Our study found a negative correlation (r=-0.37) between nutritional status and the incidence of respiratory infections, indicating that participants with malnutrition or at nutritional risk were more likely to experience respiratory tract infections. This finding is consistent with previous research, such as the study by Zheng et al. [31], which demonstrated that malnutrition impairs immune function, reduces the body’s ability to respond to pathogens, and increases susceptibility to infections such as pneumonia and influenza [32]. Malnutrition leads to decreased production of immune cells and impaired antibody responses, both of which are crucial for preventing respiratory infections [33,34]. Participants with malnutrition often have lower protein and micronutrient intake, which can compromise respiratory muscle strength. Older adults with limited mobility, chronic comorbidities, or a history of recent illness are at a higher risk of developing various infections.

The results underscore the importance of early identification and intervention for older adults at nutritional risk. Implementing nutritional support measures may help enhance immune function, reduce infection risk, and improve overall health outcomes in nursing home residents [22]. Our findings also highlight the need for regular screening and timely intervention to prevent related complications.

Limitations of the study

The main strengths of this study include the use of validated tools (MNA-SF), a clearly defined population, and a detailed regional context. This study has several limitations. A longitudinal study with a larger number of participants would certainly provide a more accurate insight into the prevalence of malnutrition and infections. Furthermore, due to the small sample size, the results cannot be generalized to the population as a whole. In addition, future research should include measures to improve nutritional status and reduce the risk of complications such as falls and respiratory infections.

## Conclusions

A high proportion of older adults in nursing homes are affected by malnutrition and nutritional risks, emphasizing the need for regular screening and timely identification of the risks of malnutrition and its consequences. Although a positive correlation between malnutrition and respiratory infections was confirmed, and no association between malnutrition and falls was found, these results emphasize the complexity of the various risk factors and the need for further research. The negative correlation between BMI and MNA-SF indicates the possibility of co-occurrence of obesity and malnutrition, which is often overlooked in daily practice. Our conclusions are directly supported by the data, emphasizing that early identification and intervention for residents at nutritional risk can significantly reduce the prevalence of malnutrition-related complications.
